# Engineering a single-chain antibody against *Trypanosoma cruzi* metacyclic trypomastigotes to block cell invasion

**DOI:** 10.1371/journal.pone.0223773

**Published:** 2019-10-16

**Authors:** Lara Maria Kalempa Demeu, Rodrigo Jahn Soares, Juliana Severo Miranda, Lisandro A. Pacheco-Lugo, Kelin Gonçalves Oliveira, Cristian Andrés Cortez Plaza, Philippe Billiald, Juliana Ferreira de Moura, Nobuko Yoshida, Larissa Magalhães Alvarenga, Wanderson Duarte DaRocha

**Affiliations:** 1 Departamento de Bioquímica e Biologia Molecular, Setor de Ciências Biológicas, Universidade Federal do Paraná,Curitiba, Brasil; 2 Departamento de Patologia Básica, Setor de Ciências Biológicas, Universidade Federal do Paraná, Curitiba, Brasil; 3 Universidad Simón Bolívar, Barranquilla, Colombia; 4 Departamento de Microbiologia, Imunologia e Parasitologia, Escola Paulista de Medicina, Universidade Federal de São Paulo, São Paulo, Brasil; 5 Centro de Genómica y Bioinformática, Facultad de Ciencias, Universidad Mayor, Santiago, Chile; 6 Faculte de Pharmacie, Universite Paris-Sud, Paris, France; Institut de recherches cliniques de Montreal, CANADA

## Abstract

*Trypanosoma cruzi* is a flagellate protozoan pathogen that causes Chagas disease. Currently there is no preventive treatment and the efficiency of the two drugs available is limited to the acute phase. Therefore, there is an unmet need for innovative tools to block transmission in endemic areas. In this study, we engineered a novel recombinant molecule able to adhere to the *T*. *cruzi* surface, termed scFv-10D8, that consists of a single-chain variable fragment (scFv) derived from mAb-10D8 that targets gp35/50. The synthetic gene encoding scFv-10D8 was cloned and fused to a 6×His tag and expressed in a prokaryotic expression system. Total periplasmic or 6xHis tag affinity-purified fractions of scFv-10D8 retained the capacity to bind to gp35/50, as shown by Western blot analyses. Pre-incubation of metacyclic trypomastigotes with scFv-10D8 showed a remarkable reduction in cell invasion capacity. Our results suggest that scFv-10D8 can be used in a paratransgenic approach to target parasites in insect vectors, avoiding dissemination of infective forms. Such advances in the development of this functional molecule will surely prompt the improvement of alternative strategies to control Chagas disease by targeting mammalian host stages.

## Introduction

American trypanosomiasis, also known as Chagas disease, is caused by the flagellate protozoan *Trypanosoma cruzi*. This parasite has been classified into six genetic groups (discrete typing units, DTUs) named TcI-TcVI, and the DTUs present substantial genetic diversity impacting on its epidemiological, biological and medical characteristics [1]. It is estimated that around 8 million people are infected with this parasite, mainly in Latin America, and it is a serious public health problem causing approximately 10,000 deaths per year [2].

Between 2000 and 2013, the most frequent transmission route of Chagas disease in Brazil was oral, followed by vectorial. Although vectorial transmission appears to be less common, probably due to the lower visibility of these cases, these figures show the persistence of vectorborne transmission even though Brazil obtained the certificate of interruption transmission by insect vector in 2006 [3–5]. Based on 2010 data, the WHO estimates that 46 cases of Chagas disease per year in Brazil are transmitted through a vector, although this is likely to be an underestimation [6]. Despite control of the main insect vector, transmission continues because other triatomine species are adapting to the home environment of human populations, and isolated foci of *Triatoma infestans* continue to exist in some states [3,7,8].

In 2015, the *Chagas Disease Epidemiological Bulletin* was published by the Ministry of Health, recording the capture of approximately 770,000 triatomines in domiciles and peridomestic areas between 2007 and 2011 [9,10]. Some studies have also shown different degrees of resistance in pesticide-resistant triatomine populations. Together, the data suggest that countries where the disease is endemic should implement alternative control methods and epidemiological surveillance [11].

The efficacy of treatments that are currently available for Chagas disease is debated owing to the side effects of many of these drugs [12]. Benznidazole resistance has been described in natural *T*. *cruzi* populations isolated from human patients, domestic vectors and sylvatic reservoirs or vectors, including parasites that have never been exposed to the drug [13,14]. Therefore, it is necessary to develop strategies to block parasite transmission in addition to new drugs.

The development of surface-binding molecules to target pathogens is key to improve drug treatments and reducing parasite transmission. As shown in *Trypanosoma brucei* (which causes sleeping sickness), a single monomeric variable antibody domain derived from camel antibodies, known as a nanobody, that targets conserved cryptic epitopes from variant surface glycoproteins can be efficiently conjugated to nanoparticles loaded with pentamidine or human trypanolytic factor to actively target trypanosomes [15–17]. The conjugation of a nanobody to drug-filled nanoparticles resulted in a 100× reduction of the IC_50_ of the drug and efficacy *in vitro* and *in vivo* against a pentamidine-resistant cell line [17]. Notably, some nanobodies can have trypanolytic activity by themselves [18].

Surface-binding polypeptides such as nanobodies or single-chain variable fragments (scFvs), which are engineered molecules derived from monoclonal antibodies (mAbs), may potentially be used as foreign genes to exploit insect microbiota as antipathogen molecules, improving the control of vector-borne diseases. This approach is known as paratransgenesis [19]. The manipulation of bacterial symbionts such as *Sodalis glossinidius*, and *Rhodococcus rhodnii* and *Corynebacterium sp*., from *Glossina sp*. (dipterans that transmit *T*. *brucei*) or *T*. *infestans* and *Rhodnius prolixus* (hemipterans that transmit *T*. *cruzi*) respectively, to express lytic peptides, scFvs or nanobodies has shown the potential to control parasite transmission [20–23].

We attempted to generate an scFv to target the *T*. *cruzi* cell surface based on the previously described mAb-10D8 [24], which targets the gp35/50 of different *T*. *cruzi* strains. Also named TcSMUG S, gp35/50 is expressed in the insect-dwelling stages of the *T*. *cruzi* lifecycle, including in infective metacyclic trypomastigotes (MTs) [25,26]. This small mucin-like protein binds to target cells via receptors and induces bi-directional Ca^2+^ signalling, which may contribute to MT cell invasion [27]. MAb-10D8 recognises epitopes containing galactofuranose residues commonly present in isolates of the TcI group [27], though these glycotopes were found in the gp35/50 glycans from Tulahuen strain (TcVI) [28]. Treatment of trypomastigote-infected mice with mAb-10D8 or its Fab fragments reduced virulence, suggesting that this mAb decreases parasite performance during acute infection [24]. The small size of scFvs favours some aspects of pharmacodynamics, and they are more easily expressed alone or fused to other polypeptides using prokaryotic systems [29–31]. In the present work, we developed a functional recombinant single-chain antibody that targets *T*. *cruzi* gp35/50 and attenuates cell invasion.

## Materials and methods

### Sequencing of mAb-10D8 light and heavy variable chains

First, 5.3 × 10^6^ cells from a secretory hybridoma of mAb-10D8 [24], were used for RNA extraction using TRIzol (Invitrogen). Following the manufacturer’s recommendations, 3 μg RNA were used for cDNA synthesis with the ThermoScript RT-PCR system (Invitrogen). Part of the light and heavy chain genes of mAb-10D8 were amplified from the cDNA according to the protocol described by Fields et al. [32]. For heavy chain amplification, a single PCR reaction using the primers VhRevU and VhForU was required. For amplification of the light chain, a VkForU universal primer was individually combined with a set of nine reverse primers, VkRev1–9, to identify the best pair for amplification of this chain. Each of the amplified chains was cloned into a pGEM-T-Easy vector and then sequenced (5 clones for VL and VH).

### *In silico* analysis of scFv-10D8

The mAb-10D8 light and heavy chain sequences were aligned according to the International ImMunoGeneTics Information System (IMGT/DomainGapAlign) standards. Alignment data were used to predict hypervariable regions (CDRs or antibody combining sites), and a two-dimensional representation of the structure, or ‘pearl necklace’, was obtained with the IMGT/Collier de Perles tool using the CDR prediction results. The three-dimensional structure was predicted using the Rosetta Web Server (GrayLab at Johns Hopkins University Baltimore, Maryland) [33]. The structures were visualised using PyMOL software (The PyMOL Molecular Graphics System, Version 2.0 Schrödinger, LLC). Models of interactions between scFv-10D8 and various glycans molecules, such as galactopyranose and galactofuranose, were obtained using *Hex* Protein Docking 8.0 software [34]. The probable amino acids that participate in binding at the interaction site and the degree of reliability of this prediction were determined using the IntFOLD programme (Integrated Protein Structure and Function Prediction Server) [35].

### Expression and extraction of the periplasmic and cytoplasmic contents of bacteria expressing scFv-10D8

A pET22b vector was used for heterologous expression of proteins in *E*. *coli*, since it directs the expression of the protein of interest to the periplasm, which is an ideal environment for acquisition of the correct conformation, using a pelB leader sequence. Based on *in silico* analysis, the sequences of the heavy and light chains were assembled in scFv format using three replicates of four glycines and one serine (GGGGSGGGGSGGGGS) as a flexible linker [32]. Based on polypeptide sequence, the synthetic gene was codon optimised for *E*. *coli* expression and the optimised sequence was synthesised, cloned into the pUC57 vector and sequenced by GenScript. This final vector was constructed by transferring the scFv-10D8 gene from pUC57-scFv-10D8 into the pET22b *Nco*I and *Not*I restriction sites (Fermentas). Expression was performed using strains of Rosetta 2 and ArcticExpress bacteria growing in Luria Bertani medium at 37°C and 180 rpm. The next day, 1:50 inocula were inoculated in 2 L culture medium and incubated at 37°C and 180 rpm until the O.D. measured 0.4–0.5 at 600 nm. Then, IPTG (0.8 mM) was added, and the cultures were again incubated in a shaker for 4 h at 37°C for Rosetta 2 cells and 12 h at 13°C for ArcticExpress cells. At the end of this period, the bacterial cells were collected by centrifugation and then resuspended in 40 mL of TES buffer (50 mM TRIS-HCl, pH 8.0, 40 mM EDTA, pH 8.0, 0.75 M sucrose) on ice, and then the osmotic shock is caused by adding 60 mL of TES buffer previously diluted 1:4 for 30 min on ice. After osmotic shock, it was centrifuged at 10000 rpm for 10 min and the supernatant recovered (Periplasm). The resulting pellet (whole bacteria without periplasm–Total extract) was suspended in 100 mL of PBS (137 mM NaCl, 2.7 mM KCl, 10 mM Na_2_HPO_4_, 2 mM KH_2_PO_4_, pH 7.2) and lysed using a sonicator (10 pulses of 1 min on ice, 30 s pause at 40% power; ultrasonic processor XL). The soluble fraction was recovered by centrifugation and the insoluble pellet resuspended in 40 mL of PBS. 10 μL of each sample (Periplasm, whole bacteria without periplasm–Total extract, soluble, and insoluble fraction) was electrophoresed on SDS-PAGE and subjected to Western blot protocol. For protein purification, the plasmid pET22-scFv-10D8 inserted into the bacterial strain ArcticExpress was again used for induction of expression following the same protocol described above in a total culture volume of 6 L. Then, the periplasmic extract (P) was concentrated by lyophilisation followed by resuspension in water and dialysation using PBS. The periplasmic extract of scFv-10D8 was purified in a 5 mL His-Trap HP column (GE Healthcare) using AKTA Protein Purification System. The recombinant protein was eluted in elution buffer containing gradient of imidazole (PBS pH 7.4 containing Imidazole 10–500 mM). Excess imidazole was removed using an Amicon Ultra-15 Centrifugal Filter Unit (MW cutoff 10 kDa), and samples were examined by SDS-PAGE and quantified by Bradford.

### Western blot analysis of scFv-10D8 expression and reactivity profile against total parasite protein extracts

For scFv-10D8 expression analysis, total protein extracts from *E*. *coli* cultures expressing scFv-10D8 were submitted to SDS-PAGE and transferred to PVDF membranes. The membrane was blocked PBS-T (Na_2_HPO_4_ 25 mM, NaH_2_PO_4_ 10 mM, pH 7.4, Tween 20 0.3%) plus 5% non-fat milk and incubated with anti-histidine primary antibody (1:3000) (Bio-Rad, USA) followed by horseradish peroxidase-conjugated anti-mouse secondary antibody (1:5000) (Bio-Rad, USA).

In order to analyse the recognition profile of mAb-10D8 and scFv-10D8, the total protein extracts of 2 × 10^6^ G strain epimastigote forms were electrophoresed (SDS-PAGE) and transferred to PVDF membranes. Membrane strip was incubated with mAb-10D8 (1:3000) followed by horseradish peroxidase-conjugated anti-mouse secondary antibody (HRP) (1:5000). Additional membrane strips were incubated with periplasmic (P) or cytoplasmic (C) fractions obtained from scFv-10D8 expression and also periplasmic fraction of unrelated scFv (scFv Loxo) followed by anti-His secondary antibody (1:3000) and finally the anti-mouse antibody conjugated to HRP (1:5000). Detection was performed with an ECL Prime Western Blotting detection reagent kit (GE Life Sciences). We used a scFv derived from LiMab7, an unrelated monoclonal antibody, as negative control, named here as scFv-Loxo [36].

### Parasite culture conditions

G strain epimastigote parasites (TcI classification according to Zingales et al. [37]) were cultured in LIT medium (1% liver infusion, 68 mM NaCl, 56 mM Na_2_HPO_4_, 5 mM KCl, 5.5 mM glucose 10 μg/L, 10% FBS, 100 μg/mL penicillin-streptomycin) at 28°C and maintained in an exponential growth phase up to 2 × 10^7^ parasites/mL. To obtain metacyclic trypomastigote forms, epimastigotes were cultured and maintained in stationary phase (5–7 × 10^7^) for 2 h in triatome artificial urine (TAU) medium at 28°C and then incubated with TAU3AAG medium (TAU medium supplemented with 10 mM glucose, 2 mM aspartic acid, 50 mM glutamic acid and 10 mM proline) [38]. Cultures were maintained at 28°C for 7 days for complete differentiation.

### Mammalian cell invasion assay with MTs

*T*. *cruzi* G strain MTs were purified in diethylaminoethyl cellulose (DEAE-cellulose) columns as described by [39]. The purified parasites were pretreated with periplasmic fractions of scFv-10D8-expressing cells in different dilutions (1:2, 1:5, 1:10, 1:20) or affinity-purified scFv-10D8 (0.25 and 2.5 μg) for 2 h at room temperature. For the invasion assay, LLC-MK2 cells were seeded in 13-mm coverslips in sterile 24-well plates at a concentration of 1 × 10^5^ cells per well and incubated overnight in RPMI medium supplemented with 10% FBS at 37°C and 5% CO_2_. The MTs pre-incubated with scFv-10D8 (periplasmic fraction or purified protein) were added to cells in a parasite/cell ratio of 100:1 and incubated for 2 h at 37°C and 5% CO_2_. The cells were washed twice with PBS then fixed with Bouin and stained with Giemsa, followed by slide scanning (motorised Axio Imager Z2 microscope [Carl Zeiss, Jena, DE] equipped with automated scanning VSlide [Metasystems, Altlussheim, DE]) and blind counting. On each slide, approximately 300 cells were counted in random fields differentiating between infected and uninfected cells. For mAb-10D8, the same procedure was applied with different cell dilutions (1:250 and 1:500). For the negative controls, an unrelated scFv (see Western blot section) was used at the same dilutions as scFv-10D8, and for the purified protein experiment, an unrelated scFv was used at the same concentrations as purified scFv-10D8 and an unrelated mAb [36] under the same conditions as mAb-10D8.

### Statistical analysis

*T*. *cruzi* invasion assay data were analysed by one-way ANOVA and t-test in GraphPad Prism version 6 for Windows (GraphPad Software, San Diego, California, USA). P values of ≤0.05 were considered statistically significant.

## Results

### Design and *in silico* analysis of scFv-10D8

**Fig 1:**
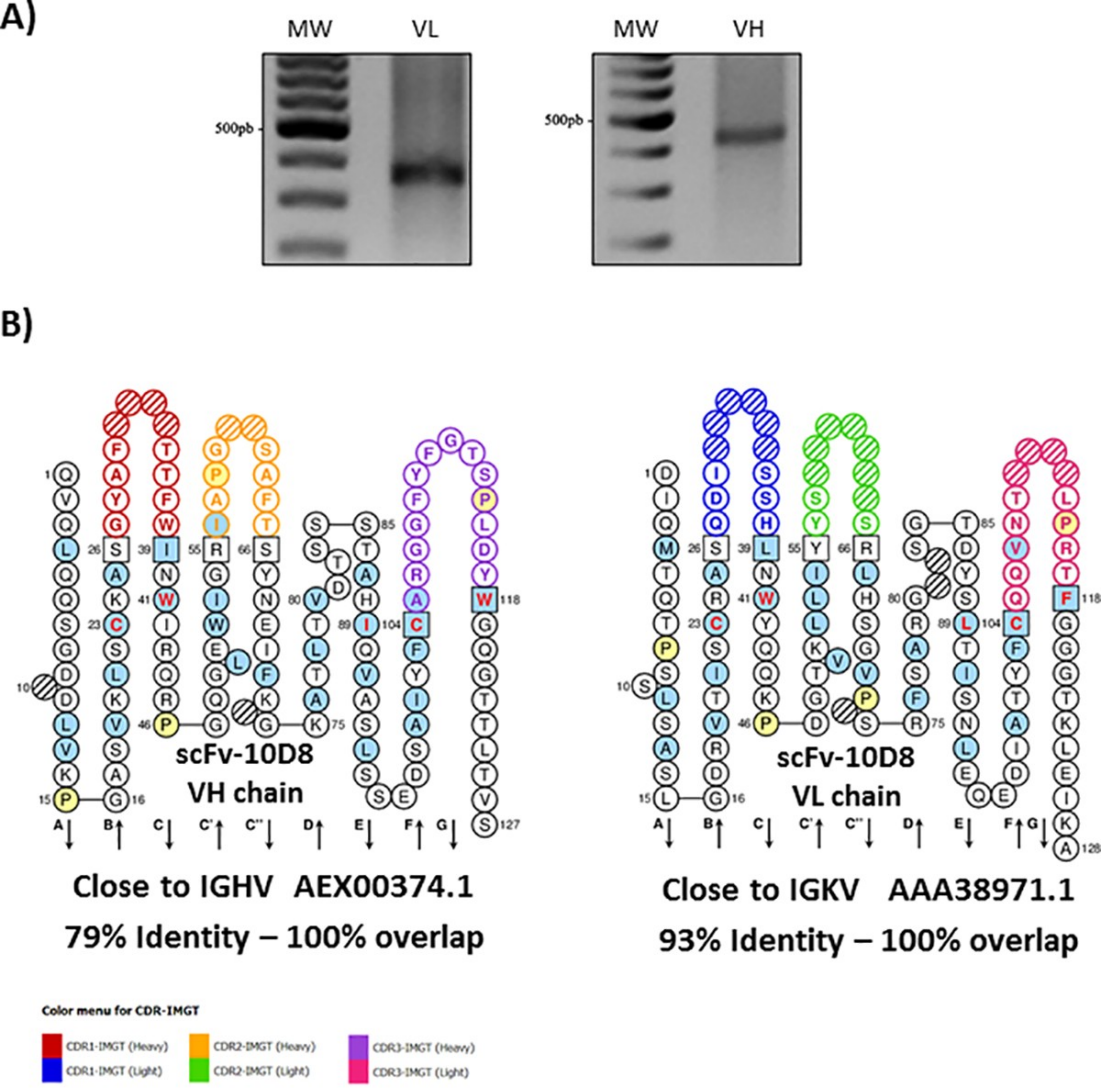
Amplification and assembly of a scFv. **a** Amplification of the light and heavy variable regions of mAb-10D8. Light variable portion amplification products are about 350 bp and heavy variable portion amplification products are about 400 bp. PCR products were obtained using cDNA as template. **b** Bidimensional representation of the scFv-10D8 (accession # MN106365) by *IMGT/Collier de Perles* highlighting the hypervariable regions (CDRs) of each variable chain. The amino acid residues were numbered according to the standard IMGT. Residues at positions 23, 41, 89, 104 and 118 are critical for the antibody structure and function. The coloured circles correspond to the CDRs of each chain, and the hatched circle indicates *gaps* that have been introduced for better alignment with the sequences of deposited variable regions.

To identify the light and heavy variable (VL and VH, respectively) domains of anti-gp35/50 mAb-10D8 IgG2b [24], cDNA from a hybridoma was subjected to a series of PCR reactions following the procedures described by Fields et al. [32]. Fig 1A shows the PCR products on agarose gels; the expected size for the light chain was approximately 350 bp, while the size of the heavy chain was expected to approach 400 bp. The PCR products were tested for aberrant chains by digestion with BciVI [32], however no such aberrant strands were found in the cDNA fragments (Data not shown), so the PCR products that met the relevant criteria were cloned and sequenced.

**Fig 2:**
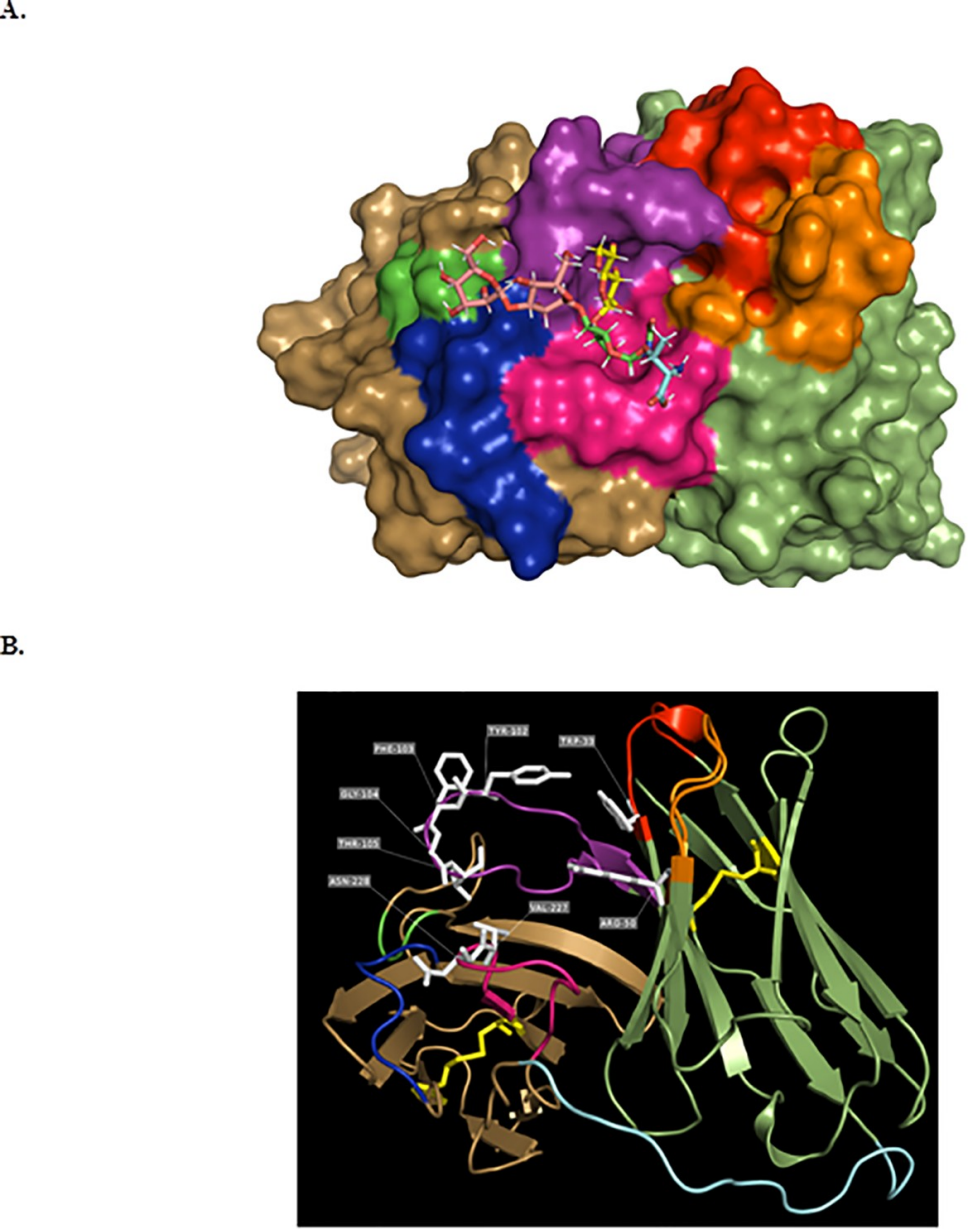
3D structure of scFv-108. Panel **a** shows the potential site of interaction between scFv-10D8 and its ligand, β-D-galactofuranose(1–4)N-acetylglucosamine. Model obtained *in silico* with the most stable interaction between the two molecules using *Hex* 8.0 software. β-D-galactofuranose(1–4)N-acetylglucosamine is represented by the sticks model, while scFv-10D8 is represented by the surface model for better visualisation of the antigen-binding site. Panel **b** represents scFv-10D8 in a VH-linker-VL format, highlighting the β-sheets, turns and flexible regions. In yellow are the cysteine residues and the disulfide bonds that are critical for the antibody structure.

The deduced amino acid sequences of the VL and VH chains were unique. Comparison with the sequences from the IMGT database allowed us to identify the closest germline mouse genes and their corresponding identity percentage, as well as complementary determining regions (CDRs), and canonical structures. The two-dimensional representations of the light and heavy chains were linked with a flexible peptide, creating the scFv-10D8 (accession number: MN106365, Fig 1B). The amino acid sequence of scFv-10D8 was also analysed using the Rosetta Web Server (GrayLab at Johns Hopkins University Baltimore, Maryland) [33], a tool capable of constructing three-dimensional models of variable antibody sequences using a combination of theoretical methods established in conjunction with the most up-to-date structural antibody information in the database. These data were analysed and visualised with PyMOL software (DeLano Scientific, San Carlos, California, USA). The resulting three-dimensional structure was similar to that of other scFvs described in the literature; it was possible to observe the formation of the antigen-binding site and the conserved disulfide bonds between cysteine residues (Fig 2A and 2B).

A structural difference between the gp35/50 of TcI group parasites and that of other groups is the presence of galactofuranose residues in the glycans of the former, which actually provide the recognition site for mAb-10D8 [27]. Given the differential recognition of mAb-10D8 in TcI group parasites, it is likely that these galactofuranose residues participate in the antibody–antigen interaction process [27]. According to Acosta-Serrano et al. [40], the gp35/50 mucin galactopyranose and galactofuranose residues are bound to N-acetylglucosamine residues at carbons 4 and 6 of this molecule, respectively. Docking assays were performed between scFv-10D8 and few potential glycans described previously, which showed more stable interactions at the antigen binding site, as expected (Fig 2A).

Since *in silico* analysis confirmed the potential of scFv-10D8 to target gp35/50, the variable chains were designed as a monomer in a VH-linker-VL format, which is the most commonly used structure using a flexible linker composed of serine and glycine. The assembled scFv-10D8 sequence was codon optimised for expression in *E*. *coli* and synthesised.

### Recombinant scFv-10D8 can bind to gp35/50 and interfere with parasite infectivity

**Fig 3:**
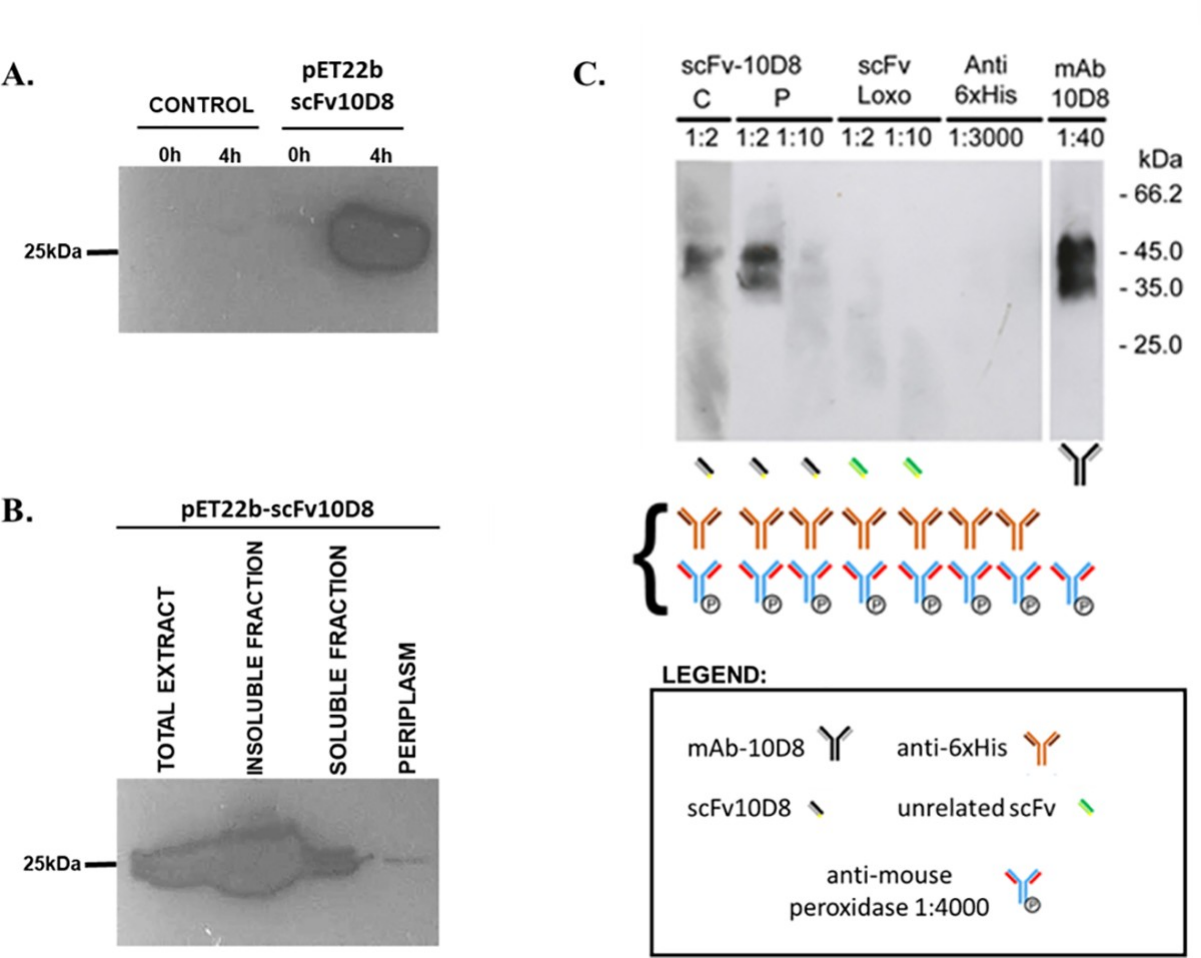
Expression, solubility and reactivity of scFv-10D8. **a** Western blotting of extracts obtained from uninduced (0 h) or induced (4 h) cultures of *Escherichia coli* Rosetta strain without (control) or with pET22b-scFv-10D8. Samples from uninduced and induced cultures were harvested, normalized by absorbance at 600 nm (cells from 1.5 mL culture at 0.5 OD are resuspended in 100 μL of sample buffer) and subjected to SDS-PAGE followed by Western blot using Anti-His antibody. **b** Bacterial cells were fractionated in periplasm, soluble cytoplasmic proteins and insoluble proteins and then tested for scFv-10D8 presence by Western blot using anti-6×His antibody. Insoluble and soluble fractions were prepared keeping the ratio during lysis process of 20 mL culture, and the total extracts corresponds to the sample from previous Western blot (4h after induction). Panel **c** shows immunoblots of epimastigote cell extracts (2×10^6^ parasites/lane) probed with scFvs or mAbs as primary antibodies and their controls. The scheme below the blots shows the sequence of antibody incubation used to detect their reactivity. Curly bracket corresponds to the antibodies used during Western blot detection step. “C” corresponds to soluble fraction of bacteria without periplasm, and “P” corresponds to a fraction containing soluble proteins located in the periplasm. Anti-6×His and an unrelated scFv (scFv Loxo) were used as negative controls and mAb-10D8 as a positive control.

To test for reactivity, scFv-10D8 was cloned into the pET22b expression vector with the aim of expressing the fusion protein at the periplasmic milieu to ensure proper folding of the scFv fused to the 6×His tag at the C-terminus. Expression assays showed an intense band compatible with the expected size in whole cell extracts (Fig 3A). However, the amount of protein directed to the periplasm was very low compared to the total and cytoplasmic fractions (Fig 3B).

The periplasmic and cytoplasmic fractions obtained after expression of scFv-10D8 were tested for the ability to recognise *T*. *cruzi* surface glycoproteins in Western blot assays. We used total protein extract from G strain epimastigote parasites with mAb-10D8 as a positive control and the periplasmic fraction of an unrelated scFv or anti-6×His antibody alone as a negative control. The scFv-10D8 extracted from the periplasm showed similar recognition pattern compared to mAb-10D8, suggesting that scFv-10D8 can also recognise gp35/50. However, cytoplasmic extract with a higher amount of scFv-10D8 did not produce a better reaction (Fig 3C). These results support the theory that the periplasm is a better environment for disulfide bond formation and thus scFv-10D8 folding. Negative controls did not show reactivity to *T*. *cruzi* proteins.

**Fig 4:**
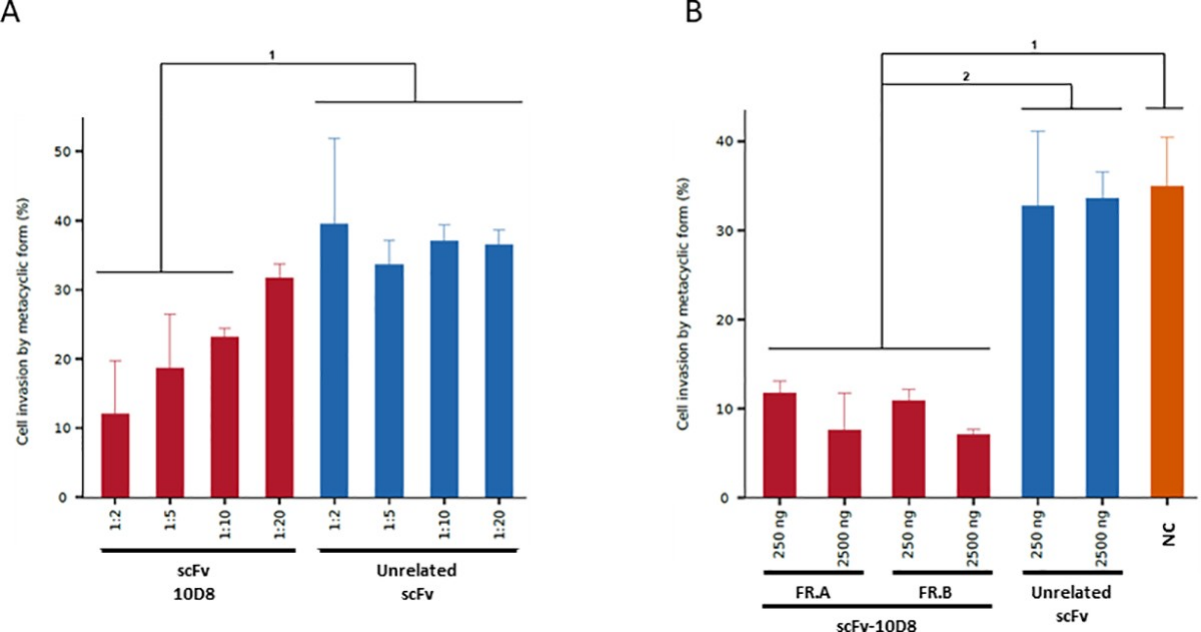
Effect of pre-incubation of metacyclic trypomastigotes (MTs) with scFv-10D8 on LLC-MK2 cell invasion. **a** MTs of *T*. *cruzi* G strain were pre-incubated with different dilutions of periplasmic fractions containing sFv-10D8 or an unrelated scFv for 2 h. **b** MTs of *T*. *cruzi* G strain were pre-incubated with different concentrations (250 and 2500 ng) for both purified protein fractions (scFv-10D8) and unrelated scFv (scFv Loxo) for 2 h and a negative control (NC, without antibody presence). FR.A and FR.B correspond to two independent fractions enriched for scFv-10D8. Statistically significant differences are represented by letters above the graph bar. The parasites were washed and incubated with LLC-MK2 cells at MOI 100:1. The percentage of infected cells was determined by the mean of blind counts of 300 cells from three independent experiments. The main statistically significant differences are indicated by horizontal bars above the columns (p ≤ 0.05).

To test whether scFv-10D8 can interfere with *T*. *cruzi* cell invasion, MTs (which also express gp35/50) were purified using DEAE-cellulose, pre-treated with different dilutions of periplasmic fraction (see S1 Fig) containing scFv-10D8 and then incubated with mammalian cells. Blind counting of noninfected/infected cells showed specific inhibition of cell invasion with scFv-10D8 compared to an unrelated scFv or mAb at different dilutions. The negative controls did not interfere with mammalian cell infection, resulting in a high infection rate (approximately 35%). However, mAb-10D8 reduced the infection rate by 3.5 fold relative to negative controls (~10%). The periplasmic fraction containing scFv-10D8 showed a dose-dependent reduction in infection rate as the dilution decreased (Fig 4A). It was observed that the results of the experiment with unrelated scFv (1:2, 1:5, 1:10 and 1:20) were significantly different to those with scFv-10D8 at 1:2, 1:5 and 1:10 dilutions (horizontal bar 1) (Fig 4A). The reduction of infection rate caused by the treatment with periplasm containing scFv-10D8 cannot be attributed to a decrease in cell viability by any bacterial components since we performed viability test demonstrating that both periplasmic fractions (unrelated scFv and scFv-10D8) caused the same percentage of MT viability at 1:2 dilution (see S1 Table).

As in the previous assay using periplasmic fractions, in the *T*. *cruzi* invasion test using distinct fractions of affinity-purified scFv-10D8 (see S1 Fig) at different concentrations (250 and 2500 ng), we observed that the infection rate was also decreased. In this assay, the infection rate in the presence of scFv-10D8 dropped 3 fold compared to the other treatment (without scFv-10D8 or unrelated scFv) reaching 12% and 7% (250 and 2500 ng scFv-10D8, respectively), which is significantly different to the results obtained from the negative controls (Fig 4B).

## Discussion

Chagas disease is a neglected tropical disease caused by *T*. *cruzi*, a protozoan parasite that undergoes morphological and biochemical changes to adapt and survive in distinct hostile environments, such as the insect gastrointestinal tract and mammalian defence mechanisms to pathogen invasion. Paratransgenesis approach using engineered ligands based on polypeptides, scFv or nanobodies to target parasite cell surface can contribute to parasite control [39,41].

The best candidates for developing *T*. *cruzi* cell surface are previously characterised mAbs because they can be humanised for use in human therapy. However, to exploit their potential, they must be engineered as recombinant molecules, such as scFvs and diabodies [42,43]. Since hybridoma cell lines are quite unstable and frequently loose the expression of functional antibody, the sequencing mAb-10D8 variable segments (VL and VH) in the present study allowed preserving the antibody expression in other systems. It is known that mAb-10D8 binds to the carbohydrate portion of gp35/50, a mucin-like protein that is expressed in the insect stages of the parasite lifecycle. Therefore, scFv-10D8 can be engineered for paratransgenesis in a similar way as has been described for nanobodies targeting *T*. *brucei* cell surface [23]. In *T*. *brucei*, De Vooght et al genetically engineered *Sodalis glossinidius*, a bacterial symbiont of *Glossina* sp., to efficiently express and release a functional trypanolytic nanobody called Nb_An46 that targets VSG [23].

The result of the IMGT/Collier de Perles shows the construction of each chain (VH-linker-VL) of the monomeric antibody in a 2D graphic representation. Based on amino acid conservation at distinct positions in the Collier de Perles representation, such as cysteine disulfide bonds, and the 3D structure prediction, we suggest that scFv-10D8 is a functional recombinant antibody. Docking analysis between scFv-10D8 and few of its potential epitopes revealed a series of probable interactions, including some interactions outside of antigen-binding regions (CDRs). However, more favourable interactions were found between CDRs and their ligands at regions that would be spatially inaccessible or non-existent in the mAb (e.g. in the space between the linker and the scFv-10D8 heavy and light chains). Data on the interactions between scFv-10D8 and glycan molecules indicate that mAb-10D8 appears to have affinity for oligosaccharides containing galactofuranose residues (data not shown), which supports previous data on the structural composition of gp35/50. The structures presented by Mendonça-Previato et al. [44] show the different assemblages of the glycidic structures in different *T*. *cruzi* strains. We observed that for *T*. *cruzi* G strain, not all coupling assays generated satisfactory results because structures that had more galactopyranose molecules blocked the physical space for galactofuranose access and increased the interaction energy. These prediction assays were also conducted using unrelated scFvs, which did not show binding capacity. However, these specific interactions must be confirmed *in vitro*.

The scFv expression analysis was performed in a pET22b+ vector, which allowed for the insertion of the pelB leader sequence for assessing expression in the periplasm, which is an environment conducive to the formation of the disulfide bonds that allow the correct conformation of proteins including scFvs [45]. The scFv-10D8 expression levels observed in the periplasm in the present study are consistent with those reported for other scFvs, which are usually low [43,46,47]. Instead of the low level of expression at the periplasm, we were able to show the binding capacity of scFv-10D8 through Western blotting, confirming the same specificity described for mAb-10D8 [48]. Here, the periplasmic expression of the recombinant protein was quite low, whatever the host strain was, but the purified scFv-10D8 was of quality good enough for an early assessment of its functionality. Expression of the scFv in the cytoplasm followed by refolding is probably a not viable alternative, since the processes are usually long, tedious and the yield of a functional recombinant protein is low. In the future, the use of genetically modified bacterial strains that have proved to be efficient for pharmaceutical development of antibody fragments will certainly improve the process [49].

A cell invasion assay in the presence of distinct molecules (mAb-10D8, scFv-10D8, and negative controls) was performed to confirm scFv-10D8 function. Accordingly, scFv-10D8 showed a clear capacity to specifically reduce parasite invasion, which is compatible with the results described by Ayub et al [43]. This result was similar to that of the experiment without purified protein. At this point, the efficiency of scFv in blocking invasion could not be compared with that of mAb-10D8 because we needed to improve the purity of mAb-10D8. Despite not being able to compare mAb-10D8 and its scFv counterpart, we can infer that the recombinant antibody maintains the mAb-10D8 ability to block the infection, even though it is a monovalent molecule. The promising results described here show that the same approach can be done for other previously described mAbs, such as mAb-2B10 that recognizes a broader range of gp35/50 [27]. The development of additional scFvs will also allow their use alone, or to create bispecific diabodies, which can improve parasite targeting.

Camara et al [50] have reported that gp35/50 is a key molecule required for parasite attachment to the internal cuticle of the triatomine rectal ampoule, thus affecting epimastigote to metacyclic trypomastigotes differentiation. These author suggest that targeting gp35/50 is appealing mechanism to block parasite transmission.

In conclusion, the present work shows the construction of an scFv capable of recognising the *T*. *cruzi* gp35/50 surface protein on Western blots and reducing mammalian cell infection by *T*. *cruzi* metacyclics. Our results in combination with published by Camara et al [50] show the great potential of using scFv-10D8 in paratransgenesis approach in a format similar to that used for *T*. *brucei*, either alone or in combination with lytic peptides to interfere with parasite::insect interaction.

## Supporting information

S1 Fig SDS-PAGE profile of purified scFv-10D8::6xHis and periplasmic fractions. 1^st^ lane corresponds to one representative enriched fraction of scFv-10D8::6xHis (Fraction A), which was obtained by affinity chromatography using His-Trap HP column (GE Healthcare). 2^nd^ lane: Periplasmic extract of scFv10D8 obtained as described in Material and Methods.
(DOCX)

S1 Table Effects of periplasmic fraction incubation on metacyclic trypomastigote viability. 5x10^6^ MTs obtained by differentiation in TAU3AAG media as previously described (Contreras et al 1988) were washed and resuspended in 100 μL of cold PBS. MTs suspensions were incubated with 100 μL of periplasmic fractions (scFv-10D8 or unrelated scFv) or PBS (negative control) for 2hs and then washed and resuspended with PBS containing Propidium Iodide (15 μg/mL). After 10 minutes of taining, the cells where washed, fixed with paraformaldehyde 2% and submitted to flow cytometry analysis. Additionally, untreated MTs were fixed in paraformaldehyde 2%, permeabilized with Triton X-100 0.05%, treated with RNase (5 ug/mL) and labelled with PI. The percentage of PI positive cells and the mean intensity of fluorescence is show below.
(DOCX)
