# Supplementary material for: Engineering a single-chain antibody against Trypanosoma cruzi metacyclic trypomastigotes to block cell invasion
Source: PLoS One. 2019 Oct 16;14(10):e0223773. doi: 10.1371/journal.pone.0223773 (PMC6795462; doi:10.1371/journal.pone.0223773)
Supplement: S1 Fig — 1st lane corresponds to one representative enriched fraction of scFv-10D8::6xHis (Fraction A), which was obtained by affinity chromatography using His-Trap HP column (GE Healthcare). 2nd lane: Periplasmic extract of scFv10D8 obtained as described in Material and Methods. (DOCX) [file pone.0223773.s001.docx]

**Supplementary material**

**Engineering a Single-Chain Antibody against *Trypanosoma cruzi* Metacyclic Trypomastigotes to Block Cell Invasion**

Lara Maria Kalempa Demeu^1^*, Rodrigo Jahn Soares^1,2^*, Juliana Severo Miranda^2^, Lisandro A. Pacheco-Lugo^1,3^, Kelin Gonçalves Oliveira^1^, Cristian Cortez^4^, Philippe Billiald^5^, Juliana Ferreira de Moura^2^, Nobuko Yoshida^3^, Larissa Magalhães Alvarenga^2^,

Wanderson Duarte DaRocha^1^**

^1^Departamento de Bioquímica e Biologia Molecular, Setor de Ciências Biológicas, Universidade Federal do Paraná–Curitiba–Paraná–Brasil.

^2^Departamento de Patologia Básica, Setor de Ciências Biológicas, Universidade Federal do Paraná–Curitiba–Paraná–Brasil.

^3^Universidad Simón Bolívar. Barranquilla, Colombia.

^4^Departamento de Microbiologia, Imunologia e Parasitologia, Escola Paulista de Medicina, Universidade Federal de São Paulo–São Paulo–Brasil.

^5^Faculte de Pharmacie, Universite Paris-Sud–France.

*Both authors have contributed equally to this work.

**Corresponding author:

Tel: (+55) 41 3361 1662

E-mail: [wandersondarocha@gmail.com](mailto:wandersondarocha@gmail.com)


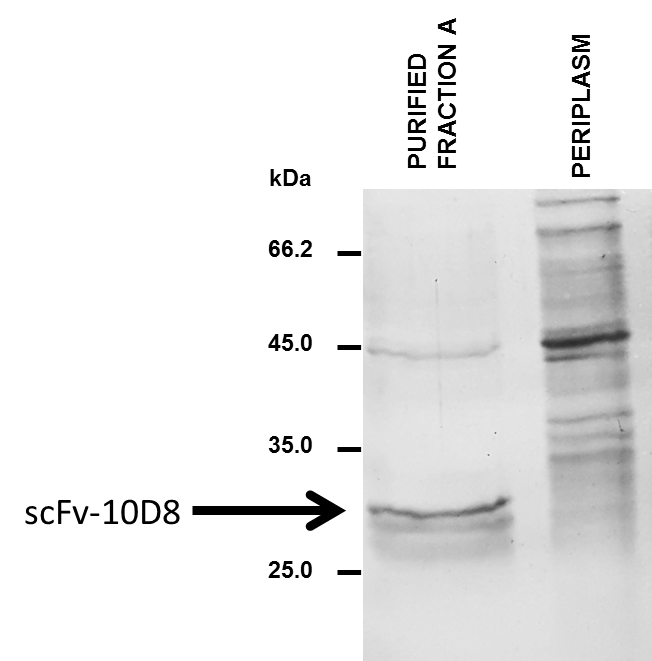


**S1 Figure: SDS-PAGE profile of purified scFv-10D8::6xHis and periplasmic fractions.** 1^st^ lane corresponds to one representative enriched fraction of scFv-10D8::6xHis (Fraction A), which was obtained by affinity chromatography using His-Trap HP column (GE Healthcare) . 2^nd^ lane: Periplasmic extract of scFv10D8 obtained as described in Material and Methods.
