# Supplementary material for: Engineering a single-chain antibody against Trypanosoma cruzi metacyclic trypomastigotes to block cell invasion
Source: PLoS One. 2019 Oct 16;14(10):e0223773. doi: 10.1371/journal.pone.0223773 (PMC6795462; doi:10.1371/journal.pone.0223773)
Supplement: S1 Table — 5x10^6^ MTs obtained by differentiation in TAU3AAG media as previously described (Contreras et al 1988) were washed and resuspended in 100 μL of cold PBS. MTs suspensions were incubated with 100 μL of periplasmic fractions (scFv-10D8 or unrelated scFv) or PBS (negative control) for 2hs and then washed and resuspended with PBS containing Propidium Iodide (15 μg/mL). After 10 minutes of taining, the cells where washed, fixed with paraformaldehyde 2% and submitted to flow cytometry analysis. Additionally, untreated MTs were fixed in paraformaldehyde 2%, permeabilized with Triton X-100 0.05%, treated with RNase (5 ug/mL) and labelled with PI. The percentage of PI positive cells and the mean intensity of fluorescence is show below. (DOCX) [file pone.0223773.s002.docx]

**Supplementary material**

**Engineering a Single-Chain Antibody against *Trypanosoma cruzi* Metacyclic Trypomastigotes to Block Cell Invasion**

Lara Maria Kalempa Demeu^1^*, Rodrigo Jahn Soares^1,2^*, Juliana Severo Miranda^2^, Lisandro A. Pacheco-Lugo^1,3^, Kelin Gonçalves Oliveira^1^, Cristian Cortez^4^, Philippe Billiald^5^, Juliana Ferreira de Moura^2^, Nobuko Yoshida^3^, Larissa Magalhães Alvarenga^2^,

Wanderson Duarte DaRocha^1^**

^1^Departamento de Bioquímica e Biologia Molecular, Setor de Ciências Biológicas, Universidade Federal do Paraná–Curitiba–Paraná–Brasil.

^2^Departamento de Patologia Básica, Setor de Ciências Biológicas, Universidade Federal do Paraná–Curitiba–Paraná–Brasil.

^3^Universidad Simón Bolívar. Barranquilla, Colombia.

^4^Departamento de Microbiologia, Imunologia e Parasitologia, Escola Paulista de Medicina, Universidade Federal de São Paulo–São Paulo–Brasil.

^5^Faculte de Pharmacie, Universite Paris-Sud–France.

*Both authors have contributed equally to this work.

**Corresponding author:

Tel: (+55) 41 3361 1662

E-mail: [wandersondarocha@gmail.com](mailto:wandersondarocha@gmail.com)

**S1 Table: Effects of periplasmic fraction incubation on metacyclic trypomastigote viability.**

| **TREATMENT** | **% OF PROPIDIUM IODIDE POSITIVE CELLS** | **MEAN INTENSITY** |
| --- | --- | --- |
| PBS | 0.32 | 186 |
| urelated scFv (periplasmic fraction) | 4.37 | 199 |
| scFv-10D8 (periplasmic fraction) | 3.14 | 170 |
| Positive control  (Triton X-100 permeabilized cells) | 97.81 | 5225 |

5x10^6^ MTs obtained by differentiation in TAU3AAG media as previously described (Contreras et al 1988) were washed and resuspended in 100 µL of cold PBS. MTs suspensions were incubated with 100 µL of periplasmic fractions (scFv-10D8 or unrelated scFv) or PBS (negative control) for 2hs and then washed and resuspended with PBS containing Propidium Iodide (15 µg/mL). After 10 minutes of taining, the cells where washed, fixed with paraformaldehyde 2% and submitted to flow cytometry analysis. Additionally, untreated MTs were fixed in paraformaldehyde 2%, permeabilized with Triton X-100 0.05%, treated with RNase (5 ug/mL) and labelled with PI. The percentage of PI positive cells and the mean intensity of fluorescence is show below.

Contreras VT, Araujo-Jorge TC, Bonaldo MC, Thomaz N, Barbosa HS, Meirelles N, Goldenberg S (1988) Biological aspect of the Dm28c clone of Trypanosoma cruzi after metacyclogenesis in chemically defined media. Mem Inst Oswaldo Cruz 83:123–133. http://dx.doi.org/10.1590/S0074-02761988000100016
